# Cost-Effectiveness of Blood Agar for Isolation of Mycobacteria

**DOI:** 10.1371/journal.pntd.0000083

**Published:** 2007-11-21

**Authors:** Michel Drancourt, Didier Raoult

**Affiliations:** 1 Fédération de Microbiologie Clinique, Assistance Publique – Hôpitaux de Marseille, Marseille, France; 2 Unité des Rickettsies, CNRS UMR 6020, IFR 48, Faculté de Médecine, Université de la Méditerranée, Marseille, France; University of Tennessee, United States of America

## Abstract

**Background:**

*Mycobacterium* species are grown using specific media that increase laboratory cost, thus hampering their diffusion in resource-limited countries. Preliminary data suggested that versatile blood agar may be also used for mycobacterial culture.

**Methodology:**

We examined the growth of 41 different *Mycobacterium* species on 5% blood agar. Over a 24-month period we analysed isolation of mycobacteria after parallel inoculation of clinical specimens into both a reference automated system (BACTEC 9000 MB broth) and 5% blood agar slant tubes, after NaOH decontamination, and compared the cost of performing 1,000 analyses using these two techniques.

**Conclusions:**

*Mycobacterium* reference species cultured on blood agar, with the exception of *Mycobacterium ulcerans*. Inoculation of 1,634 specimens yielded 95 *Mycobacterium* isolates. Blood agar performed significantly more efficiently than BACTEC 9000 MB broth (94 vs 88 isolates, *P* = 0.03). Decontamination of *Candida albicans* in 5 specimens by addition of amphotericin B in blood agar yielded one more *M. tuberculosis* isolate that could not be isolated in BACTEC broth. Uneven distribution of time to culture positivity for *M. tuberculosis* had a median (range) of 19±5 days using blood agar and 26±6 days using BACTEC 9000 MB broth. Cost for 1,000 analyses in France was estimated to be of 1,913 euros using the blood agar method and 8,990 euros using the BACTEC 9000 MB method. Blood agar should be regarded as a first-line medium for culturing *Mycobacterium* species. It saves time, is cost-effective, is more sensitive than, and at least as rapid as the automated method. This is of particular importance for resource-limited countries in which the prevalence of tuberculosis is high.

## Author Summary

Mycobacteria are organisms responsible for animal and human infections comprising tuberculosis due to *Mycobacterium tuberculosis* and other opportunistic infections. Such infections require specific antibiotic treatment and prevention of secondary cases in the occurrence of pulmonary tuberculosis. The accurate diagnosis of mycobacteria infection is therefore of prime importance. Isolation and culture of mycobacteria from diseased clinical specimens is the gold standard for diagnosis. It relied for decades on the use of specific isolation media, resulting in most laboratories not attempting such diagnosis. Alternatively, specific automates and culture broths are available only in developed countries. We herein demonstrate that blood agar, a basic medium widely and routinely used in laboratories worldwide, is suitable for the isolation and culture of mycobacteria encountered in human pathology, including tuberculosis. It performed at least as well as reference culture broth. Morever, using blood agar was cost-effective. Blood agar should be recommended as a routine medium for the isolation of most pathogenic organisms, including mycobacteria, both in developing and developed countries.

## Introduction

Specific media, such as egg-based media (e.g. Lowenstein Jensen medium), agar-based media (e.g. Middlebrook media) and liquid media (e.g. Middlebrook and BACTEC broths) are recommended for culturing *Mycobacterium* species [1]. Such requirements pose logistic and economic problems, especially in resource-limited areas where bacteriological culture facilities are few and the prevalence of mycobacterial infections, notably tuberculosis, is high. The cost of automatic detection using reference automated mycobacterial culture systems is high. The rationale for using specific media for culturing mycobacteria was to ensure the growth of *Mycobacterium* species without supporting the growth of contaminants. However, effective decontamination procedures have been developed for non-sterile samples [1]. We therefore questioned the utility and cost-effectiveness of an alternative medium, blood-agar, for the isolation and growth of *Mycobacterium* species as it is relatively commonly available and inexpensive. Moreover, it may save time and expense by avoiding duplicate inoculation for sterile specimens when other microorganisms are suspected. Sporadic papers have reported the isolation of *Mycobacterium tuberculosis* on standard blood-agar incubated for 3 weeks or more [2],[3] and proof-of-principle studies have been published [4],[5]. However, it has not been evaluated whether blood agar is useful for the isolation of opportunistic non-tuberculous *Mycobacterium* species, what the contamination frequency is for non sterile samples, and what the cost-effectiveness of such a diagnostic approach is.

The data presented herein show that ordinary blood agar medium can support the growth of *Mycobacterium* species other than *Mycobacterium ulcerans*, in a cost-effective way and that contamination is rarely a problem if decontamination procedures are strictly followed.

## Methods

### *Mycobacterium* strains

Forty-one reference strains of *Mycobacterium* species (Institut Pasteur, Paris, France and American Tissue Culture Collection, Rockville, Maryland) were tested (Table 1). An aliquot of 10 µl of stock culture (concentration 10^2^ colony-forming units/ml) was streaked simultaneously onto Middlebrook 7H10 agar (Becton Dickinson Diagnosis Systems, Le Pont de Chaix, France) and blood-agar slant tubes containing 21 g/l peptones, 1 g/l starch, 5 g/l NaCl, 12 g/l agar and 5% defibrinated sheep blood, pH 7.3 (Bio Technologie Appliquée, Dinan, France). The agar slants so inoculated were incubated for 28 days. Each culture was performed in triplicate.

**Table 1:** Growth of 40 *Mycobacterium* spp. reference strains (alphabetical order) on Middlebrook agar and sheep-blood agar.

Days for colonies to be visible on				
Mycobacterial species	Strain	Blood agar	Middlebrook agar	Growth temperature (°C)
*M. abcessus*	CIP 104536^T^	1	1	37
*M. aichiense*	ATCC 27280	7	7	37
*M. aurum*	CIP 104465^T^	1	1	37
*M. avium* subsp. *avium*	CIP 104244^T^	9	9	37
*M. bohemicum*	CIP 105811^T^	6	6	37
*M. bolletii*	CIP 108541^T^	6	6	37
*M. branderi*	CIP 104592^T^	6	6	37
*M. chelonae*	CIP 104535^T^	11	14	37
*M. conspicuum*	CIP 105165^T^	9	9	37
*M. fallax*	CIP 8139^T^	3	3	30
*M. flavescens*	CIP 104533^T^	3	3	37
*M. fortuitum*	ATCC 49404	2	2	37
*M. fortuitum subsp F*	ATCC 49403	2	2	37
*M. fortuitum subsp F*	CIP 104534^T^	2	2	30
*M. gordonae*	CIP 104529^T^	9	6	30
*M. haemophilum*	CIP 105049^T^	9	No growth	30
*M. heidelbergense*	CIP 105424^T^	9	9	37
*M. hiberniae*	CIP 104537^T^	9	9	37
*M. imunogenum*	ATCC 1066845	6	7	37
*M. interjectum*	ATCC 51457	6	6	37
*M. intermedium*	CIP 104542^T^	6	6	37
*M. intracellulare*	CIP 104243^T^	14	6	37
*M. kansasii*	CIP 104589^T^	15	9	37
*M. mageritense*	CIP 104973^T^	6	6	37
*M. marinum*	CIP 104528^T^	6	6	30
*M. mucogenicum*	ATCC 49650	6	6	37
*M. mucogenium*	CIP 105223^T^	2	2	30
*M. novocastrence*	CIP 105546^T^	6	6	37
*M. parafortuitum*	ATCC 19686	3	3	37
*M. peregrinum*	CIP 105382^T^	9	10	37
*M. phlei*	CIP 105389^T^	1	1	37
*M. septicum*	ATCC 700731	2	2	37
*M. simiae*	CIP 104531^T^	6	6	37
*M. smegmatis*	CIP 104444^T^	1	1	37
*M. szulgai*	CIP 104532^T^	16	11	37
*M. terrae*	CIP 104321^T^	6	6	37
*M. ulcerans*	CIP 105425^T^	No growth	14	37
*M. vaccae*	CIP 105934^T^	2	2	37
*M. wolinsky*	CIP 106348^T^	6	6	30
*M. xenopi*	CIP 104035^T^	24	16	37

The number of days before colonies became visible is indicated for each species on each growth medium. ATCC, American Tissue Culture Collections; CIP, Collection de l'Institut Pasteur.

### Clinical specimens

The study was approved by the local ethical committee according to French laws. Between January 2005 and December 2006, the sensitivity and contamination rate of cultured *Mycobacterium* species from clinical specimens on blood agar media was prospectively evaluated by parallel inoculation of eligible specimens into BACTEC 9000 MB broth. Eligible specimens comprised Ziehl-Neelsen positive respiratory tract specimens and all other specimens except cerebrospinal fluid, blood and bone marrow regardless of the Ziehl-Neelsen staining result. Prior to inoculation, respiratory and faecal samples were decontaminated as described previously [6]. In brief, an average 1 ml of contaminated specimen was mixed with 1 ml of decontamination solution (NaOH, 2% w/vol final concentration; N-acetyl-L-cystein, 0.5% w/vol final concentration) by gentle vortexing for 15 seconds and incubated at room temperature for 15 min. After gentle vortexing, 48 ml of phosphate buffered saline (PBS) pH 6.8 (BioMérieux, La Balme les Grottes, France) were added and the suspension was centrifuged at 2,8449 g for 20 minutes. The supernatant was discarded and the pellet was resuspended into 5 ml sterile PBS; 2 ml were inoculated into a BACTEC 9000 MD broth bottle and 0.2 ml were inoculated onto blood agar. The samples obtained from cutaneous and lymph node biopsies were crushed aseptically into Penta mixture (Becton Dickinson Diagnostic Systems) and sterile isotonic water, respectively. All samples were inoculated in parallel into BACTEC 9000 MD broth (Becton Dickinson Diagnostic Systems) as well as onto 5% sheep blood agar in slant tubes incubated at 37°C and 30°C for skin biopsy specimens. Time of positive detection in the BACTEC system and time to grow colonies on blood agar as monitored by naked eye examination of tubes three times a week, were observed for 8 weeks. Isolates were identified after Ziehl-Neelsen staining by phenotypic analyses and partial *rpo*B gene sequence analysis [7],[8].

### Economic evaluation

We compared the cost of inoculation onto sheep blood agar in tube and into the BACTEC system by incorporating the cost of blood agar in tube, the cost of either BACTEC system or incubator with a 5-year period for depreciation and the averaged cost of labour on the basis of 2006 salary in France. We also evaluated the cost of labour for blood agar-based technique in Algeria, Brazil, India, Laos and Malawi using 2006 salaries.

### Statistical analyses

Numerical variables were compared using the Fisher's exact test.

## Results

### *Mycobacterium* strains

*M. ulcerans* grew only on Middlebrook 7H10 agar and *M. haemophilum* grew only on blood agar media. All other *Mycobacterium* species tested grew equally well on both blood agar and Middlebrook 7H10 agar media (Table 1). Compared to those observed in blood agar media, *Mycobacterium gordonae*, *Mycobacterium szulgai*, *Mycobacterium xenopi*, and *Mycobacterium intracellulare* grew more rapidly on Middlebrook 7H10 agar. *Mycobacterium chelonae* grew more rapidly on blood agar. Results were identical in triplicate experiments.

### Clinical specimens

A total of 7,419 clinical specimens submitted for the isolation and culture of mycobacteria during this 24-month period yielded 156 *Mycobacterium* isolates (prevalence = 2.1%) comprising 118 *M. tuberculosis* complex isolates and 38 non-tuberculous isolates. 1,634 clinical specimens eligible for and included in the present study yielded 95 *Mycobacterium* isolates (prevalence = 5.8%) (Table 2) including 84 *M. tuberculosis* organisms isolated from 48 samples obtained from the respiratory tract, 27 from lymph nodes, 7 from biopsies and 2 from stools. Sixteen M. tuberculosis (19%) isolates resistant to streptomycin included two multi-drug resistant (MDR) (2.4%) isolates and no extensively drug-resistant isolate. A total of 5 *M. avium* isolates were cultured from 3 samples obtained from the respiratory tract, 1 from lymph node and 1 from stools. *M. xenopi* was cultured from the respiratory tract of one patient and a knee prosthesis abscess in another patient. *M. marinum* was cultured from a skin biopsy in one patient. *M. fortuitum* was cultured from a bone biopsy in one patient. *M. chelonae* and *Mycobacterium massiliense* were cultured from respiratory tract specimens in one patient each. Blood agar inoculation missed one *M. tuberculosis* complex isolate (1%) which grew from a lymph node after 32-day inoculation in BACTEC broth only. BACTEC broth inoculation missed 7 *Mycobacterium* organisms (7.3%) which were isolated on blood agar only and included 6 *M. tuberculosis* complex and 1 *M. marinum* organisms. The number of missed *M. tuberculosis* isolates was significantly higher with the BACTEC than the blood-agar technique (*P* = 0.03). *M. tuberculosis* complex isolates missed by the BACTEC inoculation included 4 isolates from lymph nodes, 1 from a lung biopsy and 1 from a respiratory tract sample contaminated with Candida albicans. Indeed, 5 respiratory tract specimens were found to have been contaminated (contamination rate of respiratory specimens, 1.5%) with *Candida albicans* on both tested media. One of these specimens was inoculated again on blood agar media in the presence of amphotericin B (16 mg/ml) and grew *M. tuberculosis*. The median time to culture positivity of M. tuberculosis was 19±5 days (range: 3–45) using blood-agar and 26±6 days (range: 7–39) using reference automated mycobacterial culture (*P* = 0.1). No difference was observed in culturing streptomycin-resistant, MDR and antibiotic-susceptible M. tuberculosis isolates.

**Table 2:** Comparative analysis of reference BACTEC and blood-agar for the isolation of mycobacteria from clinical specimens.

			BACTEC	Blood-agar	*P*
Specimens					
	Respiratory tract		328	328	1
	Other specimens		1,306	1,306	1
	Total		1,634	1,634	1
Isolates					
	Respiratory tract				
		*M. tuberculosis* (n = 48)	46	48	0.2
		Other species (n = 6)	6	6	1
		Total (n = 54)	52	54	0.2
	Other specimens				
		*M. tuberculosis* (n = 36)	32	35	0.1
		Other species (n = 5)	4	5	0.5
		Total (n = 41)	36	40	0.1
	All specimens				
		*M. tuberculosis* (n = 84)	78	83	0.06
		Other species (n = 11)	10	11	0.5
		Total(n = 95)	88	94	0.03
Contamination rate (respiratory specimens)			1.5%	1.5%	1
Delay for isolation (days)			22±6	19±5	0.1

### Economic evaluation

In France the annual cost for performing 1,000 BACTEC analyses including incubator/alert machine for 2,092 €, reagents for 6,090 € and labour for 78 € gave a total of 8,990 €. For the blood-agar method the annual cost for performing 1,000 analyses included one incubator for 57 €, reagents for 1,076 € and labour for 780 € gave a total of 1,913 €. In other countries, the estimated labour cost for performing 1,000 analyses using blood agar ranged from 37 € in Algeria and Brazil, 40.5 € in Malawi, 45 € in India and Laos and 50 € in Romania. The cost of reagents is variable, in many laboratories (such as in Laos), farm animal**s** are blood sampled by technician**s** to generate blood agar at very low cost.

## Discussion

This study extends the findings of previous anecdotal [2], [3]–[9] and proof-of-principle studies [4], [5]–[13] and demonstrates that blood-agar in slant tubes outperforms the reference automated method for isolation of mycobacteria from clinical specimens. Most *Mycobacterium* species encountered in the clinical microbiology laboratory readily grew onto 5% sheep-blood agar, except for *M. ulcerans*, a fastidious organism which is rarely isolated from diseased tissues in patients with Buruli ulcer [14]. The characterisation of adverse factors preventing this species culture on blood agar was beyond the scope of this study. These data extend previous information to a number of *Mycobacterium* species routinely encountered in microbiological laboratory practice, regardless of whether the species were slow or rapid growers. Previous study indeed demonstrated that blood-agar was at least equivalent to egg-based medium for the isolation of *M. tuberculosis* from respiratory and lymph node specimens [5]. In present study, the sensitivity of blood agar for culturing *Mycobacterium* isolates from clinical specimens was 98.9% (1/95 isolate was cultured in the BACTEC bottles only). This *M. tuberculosis* isolate had been recovered from a diseased lymph node and there was no obvious reason for the lack of growth on blood agar. No clinical material was left in order to reproduce the parallel inoculation. Seven *M. tuberculosis* isolates made on blood-agar medium failed to grow in BACTEC bottles, thus giving a significantly lower sensitivity (92.6%) of the BACTEC medium compared to blood agar for the isolation of mycobacteria. *M. marinum* grew in blood agar incubated at 30°C and not in BACTEC incubated at 37°C. This observation agrees with the reported optimal growth temperature of this species and that some *M. marinum* isolates grow better on blood than on Middlebrook agar [15]. Blood agar slants offer the possibility of incubation at different temperatures whereas all automatic systems, including BACTEC, do not allow for such modulation. We suggest that the *M. tuberculosis* complex isolates which did not grow in BACTEC bottles, failed to grow as the inocula were too small to promote growth in the 40-ml volume of broth but large enough to promote minute growth on solid medium. The contamination frequency of 1.5% on blood agar for respiratory tract specimens was low and warrants further evaluation in other laboratories. We used amphotericin B to decontaminate samples inoculated on blood agar, whilst amphotericin B cannot be used in BACTEC system as it interferes with the detection system. Accordingly, one respiratory tract *M. tuberculosis* isolate was made on blood agar after amphotericin B decontamination.

The median time to culture positivity for *M. tuberculosis* was shorter, albeit non-significantly, for blood-agar than for the automated mycobacterial culture. In our experience, the slant tube format prevented the otherwise rapid desiccation of blood-agar and allowed long-term incubation of the clinical specimen at 37°C. It also minimized the risk of infection for the laboratory workers [16]. These data indicate that blood-agar in slant tube**s** is a suitable alternative medium for the routine isolation of mycobacteria for clinical specimens.

Our economic analysis demonstrated that, at least in France, blood-agar was 4.7 times less expensive than a reference automated mycobacterial culture (BACTEC broth) system. We estimated a cost of 1.9 € per specimen for the blood-agar method. For developing countries we estimated a labour cost ranging from 37 € to 50 € for 1,000 analyses. Demonstration of the cost-effectiveness of blood-agar slants for the secure isolation of mycobacteria is of particular importance for the management in resource-limited countries where mycobacterial infections, chiefly tuberculosis, are highly prevalent. The microscopic-observation drug-susceptibility (MODS) assay has recently been demonstrated to be a cost-effective assay for *M. tuberculosis* isolation in Peru, costing an estimated 2 US$ per specimen [17]. The MODS assay simply relies on low-power microscopic examination of inoculated liquid medium and median time to culture positivity was significantly shorter for MODS than for the automated mycobacterial culture. MODS, unlike blood agar slants, however requires specific training [17].

Beyond standard laboratory equipment, automated mycobacterial culture requires computer-linked automated culture incubators and the servicing of sophisticated equipment which may not be easily achieved in resource-limited countries. Indeed, as for exemple, CSF specimens only are routinely cultured for M. tuberculosis using egg-based medium in Laos (P. Newton, personnal communication). The blood-agar method requires no specific equipment and the same tube allows for the isolation of non-mycobacterial bacteria and mycobacteria, thus even reducing the cost per analysis that we estimated. Contrary to the MODS assay, labour costs were not found to be significantly higher for the blood-agar method than the automated BACTEC method. Blood-agar method requires only an incubator, basic equipment in clinical laboratory and aseptically collected mammalian blood and NaOH which can be readily obtained in any country. This is of concern for resource-limited countries where blood-agar, but not specialized media, are available and mycobacterial infections are highly prevalent. In addition, developed countries require simplified procedures for the broadest spectrum of isolation and growth of organisms. Blood agar is a versatile medium routinely used in the laboratory for the isolation of both rapidly growing and fastidious bacteria from most clinical specimens. Prolonged incubation on blood agar has been advocated for the isolation of *Bartonella* species from clinical specimens such as lymph node**s** and mycobacteria including *M. tuberculosis* appeared to be the first group of organisms to be isolated from such diseased specimens [18].

This study suggests that the isolation of mycobacteria on blood agar is not an anecdotal finding and that contamination of culture is not a frequent problem. We submit that if incubated for sufficiently long time ordinary blood agar is a cost-effective method for culturing *Mycobacterium* species from various clinical specimens. Blood agar was not only inexpensive, but was also more sensitive and as rapid as the reference automated method used in industrialised countries.
